# Defining Quality Criteria for Nanoplastic Hazard Evaluation: The Case of Polystyrene Nanoplastics and Aquatic Invertebrate *Daphnia* spp.

**DOI:** 10.3390/nano13030536

**Published:** 2023-01-28

**Authors:** Anita Jemec Kokalj, Margit Heinlaan, Sara Novak, Damjana Drobne, Dana Kühnel

**Affiliations:** 1 Biotechnical Faculty, University of Ljubljana, Večna pot 111, 1000 Ljubljana, Slovenia; 2 National Institute of Chemical Physics and Biophysics, Akadeemia tee 23, 12618 Tallinn, Estonia; 3 Helmholtz Centre for Environmental Research—UFZ, Permoserstr. 15, 03418 Leipzig, Germany

**Keywords:** freshwater crustacean, mechanisms of action, daphnia, study quality, ecotoxicity, microplastics, nanoplastics, polystyrene nanoplastics (PSNP), quality criteria, study reporting completeness

## Abstract

Polystyrene nanoparticles are the most investigated type of nanoplastics in environmental hazard studies. It remains unclear whether nanoplastic particles pose a hazard towards aquatic organisms. Thus, it was our aim to investigate whether the existing studies and data provided therein are reliable in terms of data completeness. We used the example of *Daphnia* spp. studies for the purpose of polystyrene nanoplastic (nanoPS) hazard evaluation. First, a set of quality criteria recently proposed for nanoplastic ecotoxicity studies was applied. These rather general criteria for all types of nanoplastics and different test organisms were then, in the second step, tailored and refined specifically for *Daphnia* spp. and nanoPS. Finally, a scoring system was established by setting mandatory (high importance) as well as desirable (medium importance) criteria and defining a threshold to pass the evaluation. Among the existing studies on nanoPS ecotoxicity for *Daphnia* spp. (*n* = 38), only 18% passed the evaluation for usability in hazard evaluation. The few studies that passed the evaluation did not allow for conclusions on the hazard potential of nanoPS because there was no consensus among the studies. The greatest challenge we identified is in data reporting, as only a few studies presented complete data for hazard evaluation.

## 1. Introduction

Global plastic pollution and the detection of micro- and nanoplastic particles in almost all environmental compartments and geographic regions has fuelled research into the possible effects of these particles on environmental organisms. One specific research question is whether nanoplastic particles exert specific and/or enhanced effects in organisms in comparison to microplastics [1,2,3,4]. However, studies have reported different conclusions regarding this question. For example, a very extensive meta-analysis of existing studies indicated that nanoplastics are less toxic than microplastic, but that nanoscale polystyrene particles (nanoPS) are more hazardous than other nanoplastic particles [5]. As reviewed by ref. [1], there are also indications of size-specific effects depending on the type of organism. However, it remains to be elucidated which size classes need specific attention for hazard evaluation, and whether additional particle characteristics play a role in hazardous effects. For example, it has been pointed out that current methodological approaches of nanoPS ecotoxicity testing have pitfalls, particularly regarding the particle additive consideration and other characteristics of the particles [2,6].

Hence, the overall goal of the current study was to assess the relevance (hereafter referred to as study quality, according to Armijo-Olivo, Stiles [7]) of existing aquatic ecotoxicity data on nanoPS for the purpose of hazard characterization. For this purpose, we conducted a case study on nanoPS toxicity towards the aquatic invertebrate, *Daphnia* spp. NanoPS was selected because it is the most used nanoplastic particle in laboratory ecotoxicity studies due to its commercial availability. *Daphnia* spp. are among the most used aquatic organisms in general and specifically used for the hazard characterization of nanoPS [1]. Hence, *Daphnia* spp. were selected for this study due to the availability of quite a number of studies. Because the process of quality evaluation of the studies was quite laborious, we focused on *Daphnia* spp.

Overall, 38 studies were assessed with regard to their quality (reporting completeness) for hazard evaluation based on a previously developed set of criteria [8] by 4 independent evaluators. We aimed to shortlist studies that adequately addressed the key characteristics of the test substance, organism, and system for (re)use of data in a scientific context (“fit-for-purpose” data). Our aim was to enhance the transparency in terms of research materials, study design, and methodology, thus supporting the reuse of data. Based on a comparison of the evaluation results and a discussion, the criteria were extensively refined and specified. Afterwards, the refined criteria were once again applied to all of the studies to evaluate their quality. To add to the criteria list provided by Jemec Kokalj, Hartmann [8], mandatory, desirable, and voluntary criteria were defined, and a scoring system was implemented to determine accepted and rejected publications. Finally, a hazard evaluation was performed based on the accepted publications. Special attention was paid to mechanistic endpoints, in particular, whether their inclusion would contribute to defining the specificity of nanoPS hazard.

## 2. Materials and Methods

### 2.1. Literature Collection

For the case study, research papers reporting the effects of nanoPS on daphnids were collected. The first publication list was extracted from a science communication article on the potential effects of nanoPS on humans and the environment (https://nanopartikel.info/en/knowledge/materials/polystyrene/, accessed on 1 November 2020). A more elaborated search was then performed via the Web of Science platform using keywords related to the chosen test organism (“daphnids,” “daphnia,” “daphnia magna,” “daphnia pulex”) AND/OR test material (“polystyrene,” “polystyrene nanoparticles,” “polystyrene nanoplastics”) AND/OR endpoint (“toxicity,” “effect,” “hazard”). The last search was performed in August 2022. Studies that focused on uptake and fate of nanoPS in daphnids were excluded from the list as the presence of particles inside the organism per se does not necessarily imply hazard. All plastic-related terms we used according to the definition given in ref. [8]. The final list of studies used in the analysis is provided in Supplementary information Table S1.

### 2.2. Data Extraction and Organisation in Matrix Table

Subsequently, all studies were categorized according to the number of parameters in the matrix table (Table S1). The respective parameters were selected based on our key questions and related to the experimental set-up as well as mechanistic information on toxicological mode-of-action.

In addition to the main text, annexes and supplementary information were also thoroughly screened since these commonly contain important experimental details (i.e., toxicity assays and related methods) as well as information on particle characteristics. This allowed for an evaluation of potential connections between particle characteristics and hazard. For example, the use of sodium azide (NaN_3_) as a preservative for nanoPS suspensions was addressed, because the literature data indicated this substance was hazardous towards aquatic organisms [5,6,9]. Studies that did not provide any information on the use of NaN_3_ were also labelled since the toxic effects reported in these studies could be attributed exclusively to nanoPS as other potentially toxic substances were not disclosed. In addition, information about surface charge/functionalization was listed since this parameter is also discussed as influencing the hazard of nanoPS [10]. Second, a subset of studies evaluating mechanistic endpoints was constructed (Table S1). By considering endpoints other than apical ones, for example oxidative stress parameters or differential gene expression, we aimed to assess the potential modes of action for nanoPS. An overview matrix table (Table S1) was constructed listing relevant information for all studies, such as NaN_3_ content of nanoPS suspensions, particle surface functionalization, and type of endpoint reported (apical/mechanistic).

### 2.3. Study Evaluation, Refinement of Quality Criteria, and Scoring System

This step aimed at evaluating all the retrieved studies with regard to their quality, based on criteria developed by Jemec Kokalj, Hartmann [8]. The basis for this parameter list specific for nano- and microplastic particles was two quality evaluation approaches developed for engineered nanomaterials [11,12]. The criteria consisted of a set of parameters that should be listed in ecotoxicity studies on nanoplastics to enable judgement about the reliability of the hazard data. The initial set of criteria [8] was the first general compilation intended for micro- and nanoplastics, while it did not consider specific questions related to a particular type of organism and plastic particle. Hence, for the purpose of this study, the initial set of criteria was further refined and specified for *Daphnia* spp. and nanoPS hazard. This was performed using an iterative process after the evaluation upon information analysis by 4 independent evaluators.

The criteria were sorted according to 5 different categories: (1) polymer particle characteristics, (2) organism characterization and testing parameters, (3) sample preparation (dispersion of as prepared or delivered nanoplastics in media used for biological experiments), (4) nanoplastic characteristics in exposure medium, and (5) documentation of study results (see Table 1). Further, a scoring system was developed, defining the number of mandatory, desirable (not mandatory but important), and voluntary (not important) criteria. Subsequently, each study was evaluated for whether the criteria were met, and the score was calculated based on the number of met criteria and their relative importance. In our further assessment, we only included studies with complete traceability of the reported results based on the information provided. For further analyses, only the studies that met all mandatory criteria were used.

## 3. Results and Discussion

### 3.1. Description of Studies Retrieved and Organization in a Matrix Table

In total, 38 scientific papers were retrieved for this analysis [6,9,13,14,15,16,17,18,19,20,21,22,23,24,25,26,27,28,29,30,31,32,33,34,35,36,37,38,39,40,41,42,43,44,45,46,47,48]. As different experimental set-ups (e.g., different particle sizes, toxicity endpoints) were applied in the studies, each of these was considered an ecotoxicity data point. This resulted in 91 different ecotoxicity data points provided altogether by the reviewed 38 papers.

Regarding sample (nanoPS) preparation for toxicity testing, as indicated in the matrix table, 64% of studies did not specify whether NaN_3_ was present or removed, 18% studies published data despite this chemical being present, and 21% of studies applied steps to remove it (e.g., dialysis, ultracentrifugation). Two studies compared the results of nanoPS with and without NaN_3_ [6,9]. Regarding particle functionalization, 59% studies did not provide any data regarding this issue, 31% of studies reported amino (NH_2_) functionalization, 25% of studies reported -COOH group functionalization, and 13% reported the presence of fluorescent dyes. Reasons for the non-reporting of NaN_3_ use and surface functionalization seemed to be twofold; first there was a lack of awareness of the potential side-effects of NaN_3_ or the impact of surface functionalization, despite the bulk of data on the latter in nanomaterial hazard research [49]. Second, importantly, it is often not easy to obtain the relevant information from commercial suppliers of nanoPS particles. In addition, equipment and expertise to analyze these two characteristics might not be widely available.

**Figure 1:**
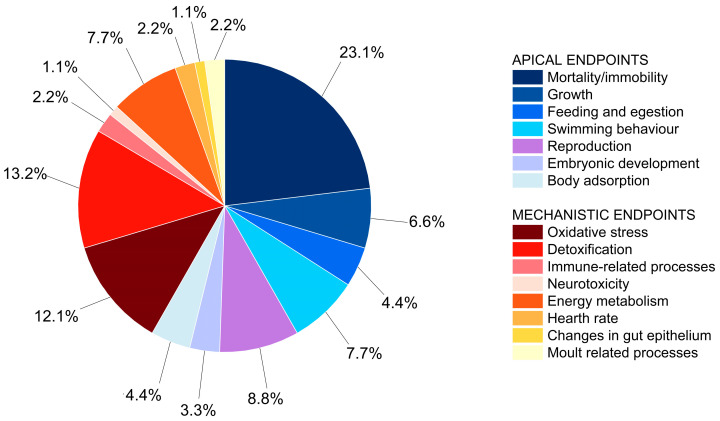
Share of ecotoxicity studies with the applied toxicity endpoints in nanoPS hazard studies using *Daphnia* spp. (*n* = 91, total number of ecotoxicity data points). Apical endpoints are shown as cold colors, mechanistic endpoints as warm colors.

Most of the studies used *D. magna* (79%), followed by *D. pulex* (18%), and *D. galeata* (3%). Different types of endpoints were assessed. Following the adverse outcome pathway concept, we classified the studies in two groups: *apical endpoints* (mortality/immobility, growth, feeding and egestion, swimming behavior, reproduction, embryonic development, body adsorption) and *mechanistic endpoints* (oxidative stress, detoxification, immune-related processes, neurotoxicity, energy metabolism, heart rate, changes in gut epithelium, moulting related processes) according to Jeong and Choi [50]. More studies assessed apical endpoints (58%; 53/91) than mechanistic endpoints (42%; 38/91) (Figure 1). Expectedly, the standard toxicity endpoint for *Daphnia* spp., mortality/immobility, was the most studied (23.7% of all endpoints) (Figure 1). Compared to other apical endpoints, 40% of studies investigated mortality/immobility, followed by reproduction (15%), swimming behavior (13%), and growth (11%) (Figure S1). Some of the toxicity endpoints, such as feeding rate, body adsorption, and swimming behavior, had specific relevance for particulate chemicals. For filter feeders such as *Daphnia* spp., physiological changes related to food intake are very relevant for nanoscale particles, as well as body adsorption e.g., ref. [18,51,52]. Additionally, physical interaction of particles is related to changes in moulting in daphnids or inflammatory changes in the gut epithelium upon ingestion [53]. Among the mechanistic endpoints, detoxification and oxidative stress were the most studied (each ~12% of all endpoints and ~30% of mechanistic endpoints). These endpoints are commonly used biomarkers for other substances as well [54]. Energy metabolism endpoints were the third most applied (18.4% of mechanistic endpoints) due to the presumed interference of particles with feeding and the energy budget [55] or lipid storage of animals [13].

In general, the endpoints used for hazard identification of nanoPS are also been commonly applied to other types of pollutants. For example, the commonly used positive control substance potassium dichromate (K_2_Cr_2_O_7_) due to its oxidative properties has also been used for evaluation of non-standardized toxicity endpoints such as swimming [56,57], feeding [58,59], heart rate [60], breathing [61], and neurotoxicity [62]. However, no data on body adsorption, growth, or embryonic development were available, as changes in these endpoints are probably not expected for this substance (Figure S2).

### 3.2. Refinement of Study Quality Criteria for Hazard Evaluation of NanoPS with Daphnia *sp.*

The criteria previously proposed for micro- and nanoplastics in general were taken as the basis for the evaluation of all nanoPS ecotoxicity studies with daphnids [8]. After an evaluation of the 38 studies by 4 independent experts, we came to the following conclusions:

(1) The initial criteria needed *additional explanation* in order to avoid misinterpretation by the different evaluators. Detailed explanations for the existing criteria elaborated in this study are given in Table 1.
(2) Some criteria needed *nanoPS property-specific clarifications*. NanoPS are usually sold as suspensions, hence some physico-properties cannot be provided (e.g., surface area) or are less commonly provided in general for nanoplastics (density, porosity, crystallography, radical production capacity, magnetic properties, surface charge, and concentration of particles prior/after the test). A common property for nanoPS commercial suspensions is the addition of NaN_3_, which acts as an antimicrobial additive. Specific attention was thus given to define criteria related to additives and impurities.
(3) Authors commonly refer to *standard guidelines* being followed for daphnids, but this was often not true in all aspects. Specifically, when the authors referred to the use of a modified standard, the study should be thoroughly checked for all methodological details. For example, the type of medium was rarely reported, but a reference to the standard was made. However, the standard itself does not define which type of test medium should be used but provides several options. Hence, each study should specify the exact type of medium used. Additionally, the standards specifically define that some media parameters, such as pH and dissolved oxygen, should be measured, but this was rarely reported. When it comes to the use of a reference chemical in the tests, this criterion should be reported in studies regardless of the standard mentioned. This is because the standards do not define the reference chemical that should be tested in each experiment.
(4) Searching for specific data in the manuscripts was *not user friendly.* We used the automatic built-in search tool in the pdf viewer whenever possible, but this was commonly not sufficient. The information in manuscripts was scattered and did not following a uniform nomenclature. Accordingly, this might have introduced a bias into the evaluation, as some criteria might have been met, but the information was not found by the evaluator. Additionally, data were available in the supplementary materials, which are not commonly the main focus of readers.
(5) The authors did not commonly provide all data related to the nanoplastics tested or the test methodology but rather referred to *previously published work*. This led to additional work to search for previously published papers and these data might be missing for proper interpretation of the toxicity data provided. Hence, a minimum set of physico-chemical properties of nanoplastics and methodological details should be available in each study. Transparency, openness, and reproducibility of research are already recognized as vital features of scientific research and data reuse aligned with FAIR principles [63]. The minimum reporting information (MRI) as identified in our study covered fundamental aspects that should be included already in research planning and later in research reporting [64]. Only more complete and transparent reporting could facilitate data search and reuse as well as study quality evaluation [65]. We suggest an MRI, as presented in Supplementary information (Table S2).

### 3.3. Classification of Criteria Importance and Threshold Development

The overarching aim of the quality assessment of studies was to provide judgement whether a study passed the evaluation, i.e., whether the data were fit for the purpose of hazard evaluation. Such a scoring system was not yet in place in Jemec Kokalj, Hartmann [8]. Hence, in order to provide a system for acceptance or rejection of studies, criteria first needed to be ranked according to their importance. We relied on a ranking elaborated previously for nanomaterials in the scope of the GUIDEnano framework [11], the DaNa project [12], and our personal experience/judgement. All the criteria that were defined as mandatory (or red questions) by these existing frameworks were also considered mandatory in the current study (Table 1). *Mandatory criteria* were those that addressed the most important aspects of the development/reporting of the study and were considered as *very relevant* for the study to be considered of acceptable quality for hazard assessment [11]. The second category included criteria with *“medium importance (desirable)”* that would enhance the quality of the study and enable advanced interpretation of the toxicity data, but were not crucial for the study to be considered acceptable. The third category included the *“not important (voluntary)”* criteria, which referred to data that were important for other engineered nanomaterials but were not as relevant for nanoPS. Following this approach, we classified the studies meeting all the mandatory criteria as acceptable for hazard assessment and the rest as not acceptable.

### 3.4. Results of Study Quality Evaluation of NanoPS Toxicity Studies with Daphnia *spp.*

**Figure 2:**
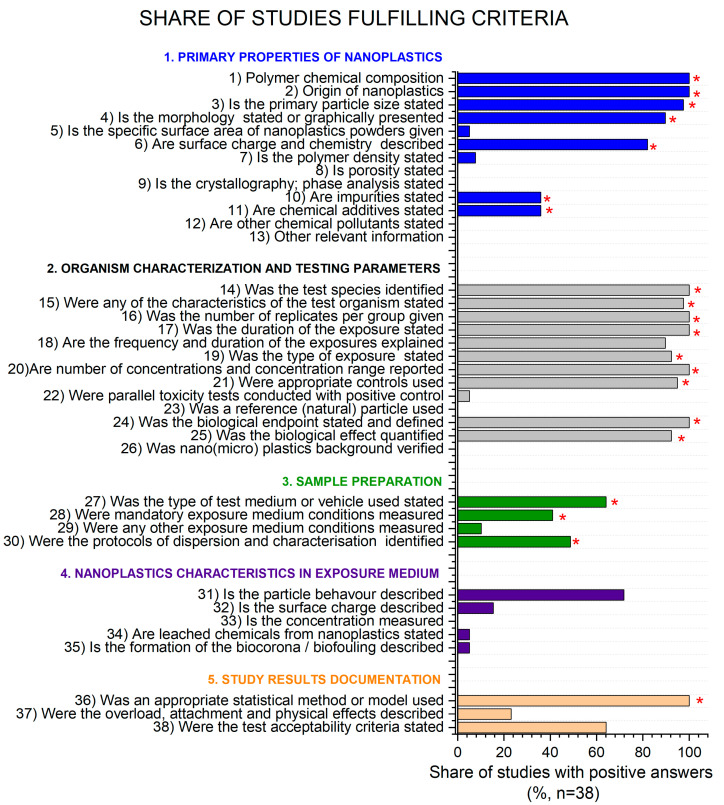
Share of studies (%) fulfilling the refined criteria. Red asterisks (*) indicate the criteria considered as very important (mandatory) according to Table 1.

The results of the quality evaluation for each individual criterion are presented in Figure 2. Most of the studies passed the criteria related to test organism characterization and testing parameters, where at least 92% of all studies fulfilled the mandatory criteria. The criteria with the least records were related to natural particle control (0%) and verification of micro/nanoplastics background (0%). It has been suggested that natural particles could be tested to evaluate whether the effects of anthropogenic particles differ from natural ones [66]. Background contamination of laboratories is needed to rule out whether the effects are indeed on the account of the nanoplastics tested [67]. Compared to organism characterization and test parameters, much lower scores were found for physico-chemical properties of nanoPS. This result was in line with a previous review [2]. The data on impurities and additives, which are considered mandatory, are a bottleneck (only 36 % of studies reported impurities or additives, respectively). The rest of the mandatory criteria in this category were fulfilled by at least 82% studies. Very few studies provided information on sample preparation; surprisingly only 64% studies defined the type of test medium and 41% reported test medium conditions (pH, oxygen). Additionally, few studies reported nanoPS properties in the exposure medium, such as particle behavior and surface charge. These, however, are not considered mandatory but desirable criteria. With regard to statistics, 100% of the studies used relevant statistical approaches to evaluate the data, but very few studies defined the specific validation criteria and their acceptability for a certain study.

Taking into consideration the threshold that all mandatory criteria needed to be fulfilled in order for the study to pass the evaluation according to Fernández-Cruz, Hernández-Moreno [11,12], only 18% of studies (7 out of 38) passed. We considered this to be a very low score and hence only 7 studies were available for further analysis of nanoPS hazard in *Daphnia* spp.

### 3.5. Hazard Evaluation based on Accepted Studies

Hazard evaluation was performed based on the accepted publications, i.e., those having met all the mandatory criteria. Among the seven studies that were classified as high-quality studies according to our refined criteria (Table 1), six studies evaluated apical endpoints [6,25,28,32,38,48]. From these studies, five studies concluded that the tested concentrations of nanoPS induced acute toxicity to *D. magna*. However, Heinlaan, Kasemets [6] emphasized that nanoPS was acutely toxic to *D. magna* only in the presence of additives (undisclosed surfactants and biocidal NaN_3_) and no more toxicity was detected once the nanoPS suspension was dialyzed. The other five studies reported the use of stabilizers in particle synthesis, however, they either concluded that the concentrations of these chemicals were kept below the toxicity thresholds of *D. magna* [25], or that they were removed before the acute toxicity assays were performed [28,32,38,48].

Mechanistic endpoints were investigated in only one high-quality study [14]. An OECD-adopted acute toxicity test with prolonged exposure of 96 h was performed. NanoPS were tested in the presence and absence of humic acid. In this study, detoxification genes were used to characterize the toxic response of neonates to the nanoPS exposure. They concluded that nanoPS exposure induced immobility and resulted in upregulation of all genes studied, while humic acid was demonstrated to have a detoxifying effect against particulate toxicity.

All in all, there was no consensus on the hazard potential of nanoPS for *Daphnia* spp. even among the comparable high-quality studies.

## 4. Conclusions

In the absence of regulatory guidance for evaluation of the environmental hazard of polymers, data quality evaluation for the selection of fit-for-purpose data has been developed. As an example, a case study of polystyrene nanoplastics (nanoPS) hazard towards aquatic model organisms, *Daphnia* spp., was conducted.

Among the reviewed 38 studies on nanoPS ecotoxicity for *Daphnia* spp., only 18% of the studies passed the evaluation for usability in hazard evaluation. Due to the low number of studies containing fit-for-purpose data for hazard evaluation, we were unable to perform a hazard evaluation for nanoPS. As shown in the quite specific case of *Daphnia* spp. ecotoxicity, it became apparent that the reporting of key characteristics of the test substance, organism, and system is essential, and the criteria probably need refinement for other test systems and organisms.

According to the criteria defined and elaborated in this study, the majority of studies are not providing sufficient information on nanoPS characteristics (e.g., surface functionalization and additive content in suspension) and the test system to evaluate the hazard of nanoPS towards *Daphnia* spp.

Based on the 7 studies that passed our evaluation, no consensus was reached regarding nanoPS hazard. Specifically, mechanistic information was scarce that would have added to our knowledge of nanoPS mechanisms of action.

Overall, this study provides the following novelties:

- Refining the existing criteria and providing an unambiguous description of each criterion for the selected case study served the purpose of re-evaluating data in scientific contexts (here: evaluation of polystyrene nanoplastic hazard);
- A scoring system and threshold values for acceptance of studies/data for the purpose of hazard evaluation were developed;
- A data reporting template for future studies, providing a list of criteria that authors can follow, was created that facilitated the retrieval of relevant information for data quality evaluation (Table S2).
- No consensus on the hazard potential of nanoPS for *Daphnia* spp. was determined, even among the high-quality studies.

## Figures and Tables

**Table 1:** Data quality criteria and relevance for hazard evaluation of impact studies on polystyrene nanoplastics (nanoPS) using *Daphnia* spp. Criteria were designed based on Kokalj et al. (2021) where the rationale for the suitability for nanoplastics is provided. The relevance of criteria was classified as mandatory (very important), desirable (of medium importance), and voluntary (not important).

No.	Criteria	Specific Guidance for Evaluator- Example for NanoPS	Relevance
**1. Primary** **Properties of Nanoplastics**			
1	Polymer chemical composition (CAS No)	This refers to the type of polymer, e.g., polystyrene (PS).	Mandatory
2	Origin of nanoplastics (commercial supply, laboratory production) including protocol of nanoplastic production and collection if relevant	Protocol for field collection is not relevant for primary nanoPS.	Mandatory
3	Is the primary particle size stated?	This refers to the diameter of particles.	Mandatory
4	Is the morphology (shape) stated or graphically presented?	This criterion is also valid if a microscopy image is provided. Most nanoPS are spheres and hence commonly not explicitly mentioned in studies.	Mandatory
5	Is the specific surface area of nanoplastic powders (e.g., BET surface) given?	This criterion is not valid for nanoPS in suspensions, because BET is measured on powders.	Desirable
6	Are surface charge and chemistry (any of the following: unlabeled, fluorescent label, functionalization, hydrophobic, hydrophilic, …)/coatings/modifications described?	Special attention should be paid to information regarding labeling. If information on labeling is provided, this is considered as information on particle modification. This criterion is also valid if the study provides information that PS NPs are unlabelled.	Mandatory
7	Is the polymer density stated?	This is not commonly reported for nanoPS.	Voluntary
8	Is porosity stated?	This is not commonly reported for nanoPS.	Voluntary
9	Is the crystallography (crystalline or amorphous phase) and phase analysis (pure or mixed) stated?	This is not commonly reported for nanoPS.	Voluntary
10	Is the concentration and/or identity of chemical impurities stated to the suspension? Or have any other steps (e.g., dialysis) been taken to remove impurities?	Impurities are chemicals that are present in plastics and/or plastic suspension but were not added intentionally. They might originate from synthesis. If the authors report that no impurities were present, this is a valid information. This criterion is also fulfilled if any step to remove impurities (e.g., dialysis, ultracentrifugation) has been undertaken.	Mandatory
11	Is the concentration and/or identity of chemical additives stated for the suspension? Or have any other steps (e.g., dialysis; ultracentrifugation) been taken to remove additives?	Additives are chemicals added to plastics and/or plastic suspension to improve their processability, properties, and performance. They are, thus, an essential part of the formulation. The most common additive of nanoPS suspension is sodium azide (NaN_3_). If the authors report that no additives were present, this criterion is also fulfilled.	Mandatory
12	Is the concentration and identity of other chemical pollutants stated?	This refers to pollutants that might sorb to nanoPS during use and disposal and is relevant only for secondary nanoPS.	Voluntary
13	Other relevant information (i.e., radical production capacity, magnetic properties for composite nanoplastics particles with magnetic substances)	This is not commonly reported for nanoPS.	Voluntary
**2. Organism Characterization and Testing Parameters**			
14	Was the test species identified?	Specific species of *Daphnia* are not required by the standard guidelines. Hence, different species can be used (*D. magna*, *D. pulex, D. galeata,* etc.)	Mandatory
15	Were any of the characteristics of the test organism (strain, sex, length, body weight, age, growth stage) stated?	Any of these characteristics should be reported, preferably at least age.	Mandatory
16	Was the number of replicates per group given?	If the test was done exactly according to the standard, it is assumed that this information was provided by referring to the standard. If use of the Daphtox kit is referenced, it is assumed that this information is provided	Mandatory
17	Was the duration of the exposure stated (e.g., 48 or 96 h)?		Mandatory
18	Are the frequency and duration of the exposures, as well as the time-points of the observation explained?	This applies to additional observations during the time of exposure.	Desirable
19	Was the type of exposure (e.g., static, flow through, diet) stated?	This is usually not specifically defined, but if medium is not changed during the exposure, this refers to a static test. In chronic tests, the reference to the standard implies feeding the daphnids.	Mandatory
20	Are the number of concentrations and concentration range tested reported?		Mandatory
21	Were appropriate controls (e.g., solvent or negative control) used?	Solvent control is usually not used with nanoPS, but negative control is.	Mandatory
22	Were parallel toxicity tests conducted with reference chemical or positive control (to ensure performance of the test system)?	This criterion is fulfilled if the authors report on the use of reference chemical. Referring to standard guideline is not sufficient, because standards do not define that the reference chemical should be tested in each experiment.	Desirable
23	Was a reference (natural) particle used (to improve interpretation of environmental relevance)?	This is not commonly reported for nanoPS.	Desirable
24	Was the biological endpoint of the toxicity study under evaluation stated and defined?	Definition of endpoint is most important when mechanistic endpoints are studied.	Mandatory
25	Was the biological effect quantified (e.g., LC50, EC50, NOEC, LOEC, 25% effect, BCF, BAF) or statistical significance determined (for mechanistic endpoints)?		Mandatory
26	Was nano(micro) plastic background verified?	This refers to background in the laboratory not in the organism.	Desirable
**3. Sample Preparation (Dispersion of as Prepared or Delivered Nanoplastic In Media Used for Biological Experiments)**			
27	Was the type of test medium or vehicle used stated?	Although the reference to the standard method is made, the type of test medium (M4, M7, Elendt, tap water) should be reported because the standard does not specifically define the type of test medium.	Mandatory
28	For ecotoxicity studies, were mandatory exposure medium conditions (at least pH and oxygen) measured? Or is this implied by mentioning the test method used, e.g., USEPA, OECD, ASTM?	Standards specifically define that some properties, pH and dissolved oxygen, should be measured. This criterion is valid if the authors mention the use of the standard guideline. The test should be done exactly according to the standard. If the standard was modified, this criterion should be checked specifically in the paper.	Mandatory
29	For ecotoxicity studies, were any other exposure medium conditions measured?	This includes hardness, conductivity, etc.	Desirable
30	Were the protocols of dispersion and characterization in the exposure medium identified? Or, were the protocols of preparation of exposure medium stated?	This includes sonication, vortexing, mixing, etc.	Mandatory
**4. Nanoplastic Characteristics in Exposure Medium**			
31	Is the particle behavior before or during the exposure described (i.e., agglomeration / aggregation / sedimentation / floating)?	This includes any type of measurement to describe the behavior of particles in exposure medium, such as DLS, nanoparticle tracking analysis, visual observation of sedimentation	Desirable
32	Is the surface charge before or during the exposure described?	This is not commonly reported for nanoPS.	Voluntary
33	Is the concentration before or during the exposure measured?	This is not commonly reported for nanoPS.	Voluntary
34	Are the concentration and identity of leached chemicals from nanoplastics under experimental conditions stated?	This is not commonly reported for nanoPS.	Desirable
35	Is the formation of the biocorona / biofouling described?	This is not commonly reported for nanoPS.	Voluntary
**5. Study Results Documentation**			
36	Was an appropriate statistical method or model used to determine toxicity?	This refers to the method used to calculate effect values or statistical difference in comparison to control. The method should take into consideration the normality and homoscedascity of data.	Mandatory
37	Were the potential overload, attachment, and physical effects in organisms described?	Usually the authors report the adsorption of particles or physical attachment.	Desirable
38	Were the test acceptability criteria stated (e.g., mortality in control must not exceed a certain percentage)? OR, were test acceptability criteria implied by mention of the test method used (e.g., USEPA, OECD, ASTM, etc)?	Standards specifically define the validity criteria. This criterion is valid if the authors mention the use of the standard guideline. The test should be performed exactly according to the standard. If the standard was modified, this criterion should be checked specifically in the paper.	Desirable

## Data Availability

Not applicable.

## Supplementary Materials

The following supporting information can be downloaded at: https://www.mdpi.com/article/10.3390/nano13030536/s1, Figure S1: Share of ecotoxicity data points; Figure S2: Distribution of apical and mechanistic endpoints for potassium dichromate; Table S1: Matrix table; Table S2: Minimum reporting information.

## References

[1] Qiao R., Mortimer M., Richter J., Rani-Borges B., Yu Z., Heinlaan M., Lin S., Ivask A. (2022). Hazard of polystyrene micro-and nanospheres to selected aquatic and terrestrial organisms. Sci. Total. Environ..

[2] Kelpsiene E., Ekvall M.T., Lundqvist M., Torstensson O., Hua J., Cedervall T. (2021). Review of ecotoxicological studies of widely used polystyrene nanoparticles. Environ. Sci. Process. Impacts.

[3] Gaylarde C.C., Baptista Neto J.A., da Fonseca E.M. (2021). Nanoplastics in aquatic systems—Are they more hazardous than micro-plastics?. Environ. Pollut..

[4] Schröter L., Ventura N. (2022). Nanoplastic Toxicity: Insights and Challenges from Experimental Model Systems. Small.

[5] Yang T., Nowack B. (2020). A Meta-analysis of Ecotoxicological Hazard Data for Nanoplastics in Marine and Freshwater Systems. Environ. Toxicol. Chem..

[6] Heinlaan M., Kasemets K., Aruoja V., Blinova I., Bondarenko O., Lukjanova A., Khosrovyan A., Kurvet I., Pullerits M., Sihtmäe M. (2019). Hazard evaluation of polystyrene nanoplastic with nine bioassays did not show particle-specific acute toxicity. Sci. Total. Environ..

[7] Armijo-Olivo S., Stiles C.R., Hagen N.A., Biondo P.D., Cummings G.G. (2010). Assessment of study quality for systematic reviews: A comparison of the Cochrane Collaboration Risk of Bias Tool and the Effective Public Health Practice Project Quality Assessment Tool: Methodological research. J. Eval. Clin. Pr..

[8] Kokalj A.J., Hartmann N.B., Drobne D., Potthoff A., Kühnel D. (2021). Quality of nanoplastics and microplastics ecotoxicity studies: Refining quality criteria for nanomaterial studies. J. Hazard. Mater..

[9] Pikuda O., Xu E.G., Berk D., Tufenkji N. (2018). Toxicity Assessments of Micro- and Nanoplastics Can Be Confounded by Preservatives in Commercial Formulations. Environ. Sci. Technol. Lett..

[10] Loos C., Syrovets T., Musyanovych A., Mailänder V., Landfester K., Nienhaus G.U., Simmet T. (2014). Functionalized polystyrene nanoparticles as a platform for studying bio–nano interactions. Beilstein J. Nanotechnol..

[11] Fernández-Cruz M.L., Hernández-Moreno D., Catalán J., Cross R.K., Stockmann-Juvala H., Cabellos J., Lopes V.R., Matzke M., Ferraz N., Izquierdo J.J. (2017). Quality evaluation of human and environmental toxicity studies performed with nanomaterials—The GUIDEnano approach. Environ. Sci. Nano.

[12] Nau K., Bohmer N., Kühnel D., Marquardt C., Paul F., Steinbach C., Krug H.F. (2016). The Dana2.0 Knowledge Base on Nanomaterials—Communicating Current Nanosafety Research Based on Evaluated Literature Data. J. Mater. Educ..

[13] Cui R., Kim S.W., An Y.-J. (2017). Polystyrene nanoplastics inhibit reproduction and induce abnormal embryonic development in the freshwater crustacean *Daphnia galeata*. Sci. Rep..

[14] Fadare O.O., Wan B., Guo L.-H., Xin Y., Qin W., Yang Y. (2019). Humic acid alleviates the toxicity of polystyrene nanoplastic particles to *Daphnia magna*. Environ. Sci. Nano.

[15] Liu Z., Cai M., Yu P., Chen M., Wu D., Zhang M., Zhao Y. (2018). Age-dependent survival, stress defense, and AMPK in Daphnia pulex after short-term exposure to a polystyrene nanoplastic. Aquat. Toxicol..

[16] Liu Z., Yu P., Cai M., Wu D., Zhang M., Huang Y., Zhao Y. (2018). Polystyrene nanoplastic exposure induces immobilization, reproduction, and stress defense in the freshwater cladoceran *Daphnia pulex*. Chemosphere.

[17] Nasser F., Lynch I. (2016). Secreted protein eco-corona mediates uptake and impacts of polystyrene nanoparticles on *Daphnia magna*. J. Proteom..

[18] Rist S., Baun A., Hartmann N.B. (2017). Ingestion of micro- and nanoplastics in *Daphnia magna*—Quantification of body burdens and assessment of feeding rates and reproduction. Environ. Pollut..

[19] Kelpsiene E., Torstensson O., Ekvall M.T., Hansson L.-A., Cedervall T. (2020). Long-term exposure to nanoplastics reduces life-time in *Daphnia magna*. Sci. Rep..

[20] Liu Z., Huang Y., Jiao Y., Chen Q., Wu D., Yu P., Li Y., Cai M., Zhao Y. (2020). Polystyrene nanoplastic induces ROS production and affects the MAPK-HIF-1/NFkB-mediated antioxidant system in *Daphnia pulex*. Aquat. Toxicol..

[21] Xu E.G., Cheong R.S., Liu L., Hernandez L.M., Azimzada A., Bayen S., Tufenkji N. (2020). Primary and Secondary Plastic Particles Exhibit Limited Acute Toxicity but Chronic Effects on *Daphnia magna*. Environ. Sci. Technol..

[22] Vicentini D.S., Nogueira D.J., Melegari S.P., Arl M., Köerich J.S., Cruz L., Justino N.M., Oscar B.V., Puerari R.C., da Silva M.L.N. (2019). Toxicological Evaluation and Quantification of Ingested Metal-Core Nanoplastic by *Daphnia magna* Through Fluorescence and Inductively Coupled Plasma-Mass Spectrometric Methods. Environ. Toxicol. Chem..

[23] Zhang F., Wang Z., Wang S., Fang H., Wang D. (2019). Aquatic behavior and toxicity of polystyrene nanoplastic particles with different functional groups: Complex roles of pH, dissolved organic carbon and divalent cations. Chemosphere.

[24] Lin W., Jiang R., Hu S., Xiao X., Wu J., Wei S., Xiong Y., Ouyang G. (2019). Investigating the toxicities of different functionalized polystyrene nanoplastics on *Daphnia magna*. Ecotoxicol. Environ. Saf..

[25] Besseling E., Wang B., Lürling M., Koelmans A.A. (2014). Correction to Nanoplastic Affects Growth of S. obliquus and Reproduction of D. magna. Environ. Sci. Technol..

[26] Chae Y., Kim D., Kim S.W., An Y.-J. (2018). Trophic transfer and individual impact of nano-sized polystyrene in a four-species freshwater food chain. Sci. Rep..

[27] Liu Z., Li Y., Pérez E., Jiang Q., Chen Q., Jiao Y., Huang Y., Yang Y., Zhao Y. (2020). Polystyrene nanoplastic induces oxidative stress, immune defense, and glycometabolism change in *Daphnia pulex*: Application of transcriptome profiling in risk assessment of nanoplastics. J. Hazard. Mater..

[28] Vaz V.P., Nogueira D.J., Vicentini D.S., Matias W.G. (2021). Can the sonication of polystyrene nanoparticles alter the acute toxicity and swimming behavior results for *Daphnia magna*?. Environ. Sci. Pollut. Res..

[29] Reynolds A., Giltrap M., Chambers G. (2019). Evaluation of non-invasive toxicological analysis of nano-polystyrene in relative in vivo conditions to D. magna. Environ. Sci. Nano.

[30] Lin W., Jiang R., Xiong Y., Wu J., Xu J., Zheng J., Zhu F., Ouyang G. (2018). Quantification of the combined toxic effect of polychlorinated biphenyls and nano-sized polystyrene on *Daphnia magna*. J. Hazard. Mater..

[31] Liu Z., Li Y., Sepúlveda M.S., Jiang Q., Jiao Y., Chen Q., Huang Y., Tian J., Zhao Y. (2020). Development of an adverse outcome pathway for nanoplastic toxicity in *Daphnia pulex* using proteomics. Sci. Total. Environ..

[32] Wu J., Jiang R., Lin W., Ouyang G. (2018). Effect of salinity and humic acid on the aggregation and toxicity of polystyrene nanoplastics with different functional groups and charges. Environ. Pollut..

[33] Grintzalis K., Lawson T.N., Nasser F., Lynch I., Viant M.R. (2019). Metabolomic method to detect a metabolite corona on amino-functionalized polystyrene nanoparticles. Nanotoxicology.

[34] Zhang W., Liu Z., Tang S., Li D., Jiang Q., Zhang T. (2019). Transcriptional response provides insights into the effect of chronic polystyrene nanoplastic exposure on *Daphnia pulex*. Chemosphere.

[35] Wu D., Liu Z., Cai M., Jiao Y., Li Y., Chen Q., Zhao Y. (2019). Molecular characterisation of cytochrome P450 enzymes in waterflea (*Daphnia pulex*) and their expression regulation by polystyrene nanoplastics. Aquat. Toxicol..

[36] Liu Z., Cai M., Wu D., Yu P., Jiao Y., Jiang Q., Zhao Y. (2019). Effects of nanoplastics at predicted environmental concentration on *Daphnia pulex* after exposure through multiple generations. Environ. Pollut..

[37] Liu Z., Jiao Y., Chen Q., Li Y., Tian J., Huang Y., Cai M., Wu D., Zhao Y. (2020). Two sigma and two mu class genes of glutathione S-transferase in the waterflea *Daphnia pulex*: Molecular characterization and transcriptional response to nanoplastic exposure. Chemosphere.

[38] Frankel R., Ekvall M.T., Kelpsiene E., Hansson L.-A., Cedervall T. (2020). Controlled protein mediated aggregation of polystyrene nanoplastics does not reduce toxicity towards *Daphnia magna*. Environ. Sci. Nano.

[39] Zhang P., Yan Z., Lu G., Ji Y. (2019). Single and combined effects of microplastics and roxithromycin on *Daphnia magna*. Environ. Sci. Pollut. Res..

[40] Fadare O.O., Wan B., Liu K., Yang Y., Zhao L., Guo L.-H. (2020). Eco-Corona vs Protein Corona: Effects of Humic Substances on Corona Formation and Nanoplastic Particle Toxicity in *Daphnia magna*. Environ. Sci. Technol..

[41] Zhang F., Wang Z., Song L., Fang H., Wang D.-G. (2020). Aquatic toxicity of iron-oxide-doped microplastics to Chlorella pyrenoidosa and *Daphnia magna*. Environ. Pollut..

[42] De Felice B., Sabatini V., Antenucci S., Gattoni G., Santo N., Bacchetta R., Ortenzi M.A., Parolini M. (2019). Polystyrene microplastics ingestion induced behavioral effects to the cladoceran *Daphnia magna*. Chemosphere.

[43] Saavedra J., Stoll S., Slaveykova V.I. (2019). Influence of nanoplastic surface charge on eco-corona formation, aggregation and toxicity to freshwater zooplankton. Environ. Pollut..

[44] De Felice B., Sugni M., Casati L., Parolini M. (2022). Molecular, biochemical and behavioral responses of *Daphnia magna* under long-term exposure to poly-styrene nanoplastics. Environ. Int..

[45] Ma C., Liu X., Zuo D. (2021). Cloning and characterization of AMP-activated protein kinase genes in *Daphnia pulex*: Modulation of AMPK gene expression in response to polystyrene nanoparticles. Biochem. Biophys. Res. Commun..

[46] Nogueira D.J., Silva A.C.d.O.d., da Silva M.L.N., Vicentini D.S., Matias W.G. (2022). Individual and combined multigenerational effects induced by polystyrene nanoplastic and glyphosate in *Daphnia magna* (Strauss, 1820). Sci. Total. Environ..

[47] Pochelon A., Stoll S., Slaveykova V.I. (2021). Polystyrene Nanoplastic Behavior and Toxicity on Crustacean *Daphnia magna*: Media Composition, Size, and Surface Charge Effects. Environments.

[48] Verdú I., Amariei G., Plaza-Bolaños P., Agüera A., Leganés F., Rosal R., Fernández-Piñas F. (2022). Polystyrene nanoplastics and wastewater displayed antagonistic toxic effects due to the sorption of wastewater micropollutants. Sci. Total. Environ..

[49] Saei A., Yazdani M., Lohse S.E., Bakhtiary Z., Serpooshan V., Ghavami M., Asadian M., Mashaghi S., Dreaden E., Mashaghi A. (2017). Nanoparticle Surface Functionality Dictates Cellular and Systemic Toxicity. Chem. Mater..

[50] Jeong J., Choi J. (2019). Adverse outcome pathways potentially related to hazard identification of microplastics based on toxicity mechanisms. Chemosphere.

[51] Botha T.L., Boodhia K., Wepener W. (2016). Adsorption, uptake and distribution of gold nanoparticles in *Daphnia magna* fol-lowing long term exposure. Aquat. Toxicol..

[52] Novak S., Kokalj A.J., Hočevar M., Godec M., Drobne D. (2018). The significance of nanomaterial post-exposure responses in Daphnia magna standard acute immobilisation assay: Example with testing TiO2 nanoparticles. Ecotoxicol. Environ. Saf..

[53] Domínguez G.A., Torelli M.D., Buchman J.T., Haynes C.L., Hamers R.J., Klaper R.D. (2018). Size dependent oxidative stress response of the gut of *Daphnia magna* to functionalized nanodiamond particles. Environ. Res..

[54] Hu M., Palić D. (2020). Micro- and nano-plastics activation of oxidative and inflammatory adverse outcome pathways. Redox Biol..

[55] Li M., Czymmek K., Huang C. (2011). Responses of *Ceriodaphnia dubia* to TiO2 and Al2O3 nanoparticles: A dynamic nano-toxicity assessment of energy budget distribution. J. Hazard. Mater..

[56] Bahrndorff S., Michaelsen T.Y., Jensen A., Marcussen L.F., Nielsen M.E., Roslev P. (2015). Automated swimming activity monitor for examining temporal patterns of toxicant effects on individual *Daphnia magna*. J. Appl. Toxicol..

[57] Nikitin O.V., Nasyrova E.I., Nuriakhmetova V.R., Stepanova N.Y., Danilova N.V., Latypova V.Z. (2018). Toxicity assessment of polluted sediments using swimming behavior alteration test with *Daphnia magna*. IOP Conf. Ser. Earth Environ. Sci..

[58] Ishii T., Kamaya M., Nagashima K. (2006). A Monitoring System of Feeding Rate in *Daphnia Magna* for Toxicity Test. J. Environ. Chem..

[59] Kamaya M., Oka Y., Fujito S., Ginatullina E. (2017). Evolution of Method for Acute Toxicity Using Fluorescent Measurement in D. Magna Feeding Suppression Bioassay in the Case of Potassium Dichromate. Int. J. Emerg. Technol. Adv. Eng..

[60] Park S., Jo A., Choi J., Kim J., Zoh K.-D., Choi K. (2019). Rapid screening for ecotoxicity of plating and semiconductor wastewater employing the heartbeat of *Daphnia magna*. Ecotoxicol. Environ. Saf..

[61] Zitova A., Cross M., Hernan R., Davenport J., Papkovsky D.B. (2009). Respirometric acute toxicity screening assay using *Daphnia magna*. Chem. Ecol..

[62] Jemec A., Tišler T., Drobne D., Sepčić K., Jamnik P., Roš M. (2008). Biochemical biomarkers in chronically metal-stressed daphnids. Comp. Biochem. Physiol. Part C Toxicol. Pharmacol..

[63] Jeliazkova N., Apostolova M.D., Andreoli C., Barone F., Barrick A., Battistelli C., Bossa C., Botea-Petcu A., Châtel A., De Angelis I. (2021). Towards FAIR nanosafety data. Nat. Nanotechnol..

[64] Higman R., Bangert D., Jones S. (2019). Three camps, one destination: The intersections of research data management, FAIR and Open. Insights.

[65] Elberskirch L., Binder K., Riefler N., Sofranko A., Liebing J., Minella C.B., Mädler L., Razum M., van Thriel C., Unfried K. (2022). Digital research data: From analysis of existing standards to a scientific foundation for a modular metadata schema in nanosafety. Part. Fibre Toxicol..

[66] Ogonowski M., Schür C., Jarsén Å., Gorokhova E. (2016). The Effects of Natural and Anthropogenic Microparticles on Individual Fitness in *Daphnia magna*. PLoS ONE.

[67] Bogdanowicz A., Zubrowska-Sudol M., Krasinski A., Sudol M. (2021). Cross-Contamination as a Problem in Collection and Analysis of Environmental Samples Containing Microplastics—A Review. Sustainability.

