# Supplementary material for: Defining Quality Criteria for Nanoplastic Hazard Evaluation: The Case of Polystyrene Nanoplastics and Aquatic Invertebrate Daphnia spp
Source: Nanomaterials (Basel). 2023 Jan 28;13(3):536. doi: 10.3390/nano13030536 (PMC9919956; doi:10.3390/nano13030536)
Supplement: Supplementary file 1 [file nanomaterials-13-00536-s001.zip › nanomaterials-2176145-supplementary.pdf]

Supplementary Materials Figures

Figure S1 Share of ecotoxicity data points

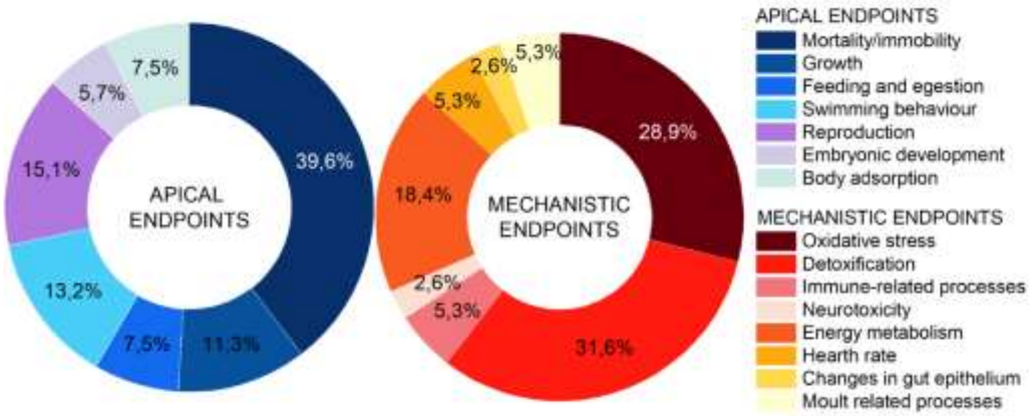

Figure S1. Share of ecotoxicity data points separately for apical endpoints (n= 55) and mechanistic endpoints (n=38). Data for all types of nanoPS (surface modification) are included.

Figure S2 Distribution of apical and mechanistic endpoints for potassium dichromate

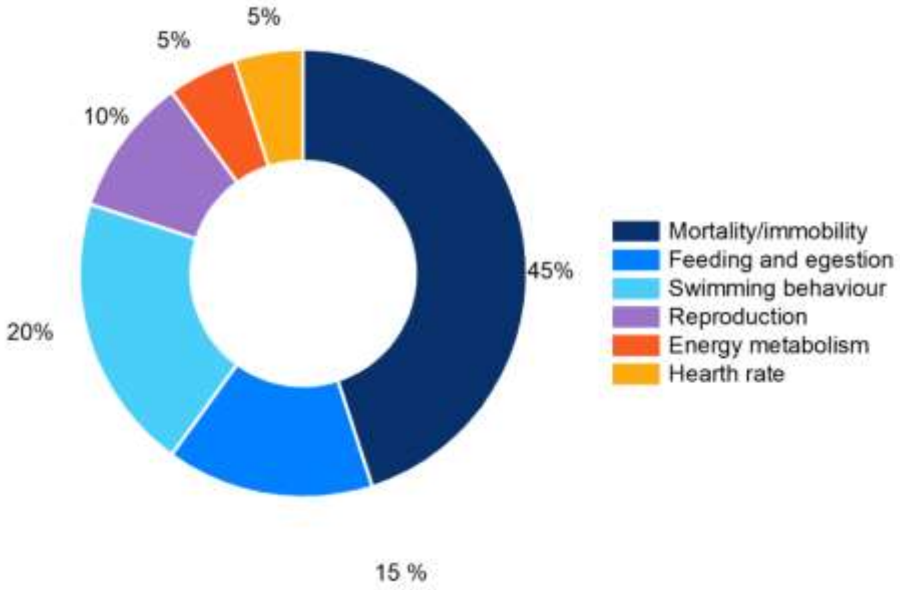

Figure S2. Distribution of apical and mechanistic endpoints for potassium dichromate (n=20). .

Table S1. Matrix table.

List of studies reporting a certain property (sodium azide, functionalisation, endpoints). The outcome of study evaluation is provided.

		Sodium Azide (NaN3)			Functionalisation					Endpoints reported		Passed the study quality evaluation
Reference		including NaN3	NaN3 removed	unspecified	NH2	COOH	unspecified	fluorescent dye	other	Apical	Mechanistic	Yes/No
Cui et al., 2017	https://doi.org/10.1038/s41598-017-12299-2	x					x			x		No
Fedare et al., 2019	https://doi.org/10.1039/c8en01457d		x		x			x		x	x	Yes
Liu et al., 2018	https://doi.org/10.1016/j.aquatox.2018.08.017			x			x			x	x	No
Liu et al., 2019	https://doi.org/10.1016/j.chemosphere.2018.09.176			x			x			x	x	No
Nasser and Lynch, 2016	https://doi.org/10.1016/j.jprot.2015.09.005			x	x	x		x		x		No
Rist et al 2017	http://dx.doi.org/10.1016/j.envpol.2017.05.048	x					x	x		x		No
Kelpsiene et al., 2020	https://doi.org/10.1038/s41598-020-63028-1		x		x	x				x		No

Liu et al., 2020	https://doi.org/10.1016/j.aquatox.2020.105420			x			x				x	No
Xu et al., 2020	https://dx.doi.org/10.1021/acs.est.0c00245	x					x	x		x	x	No
Pikuda et al. 2019	https://doi.org/10.1021/acs.estlett.8b00614	x	x			x				x		No
Vincentini et al. 2019	https://doi.org/10.1002/etc.4528		x						x	x		No
Zhang et al. 2019	https://doi.org/10.1016/j.chemosphere.2019.04.115			x	x	x					x	No
Lin et al. 2019	https://doi.org/10.1016/j.ecoenv.2019.05.036			x	x	x				x	x	No
Heinlaan et al. 2020	https://doi.org/10.1016/j.scitotenv.2019.136073	x	x			x				x		Yes
Besseling et al 2014	https://doi.org/10.1021/es503001d			x		x				x		Yes
Chae et al 2018	https://doi.org/10.1038/s41598-017-18849-y	x					x				x	No
Liu et al 2021	https://doi.org/10.1016/j.jhazmat.2020.123778			x			x				x	No
Vaz et al 2021	https://doi.org/10.1007/s11356-021-12455-2			x			x			x		Yes
Reynolds et al., 2019	https://doi.org/10.1039/c9en00434c			x			x			x	x	No
Lin et al., 2019	https://doi.org/10.1016/j.jhazmat.2018.10.056			x			x			x		No
Liu et al., 2021	https://doi.org/10.1016/j.scitotenv.2020.144249	x					x			x	x	No
Wu et al., 2019a	https://doi.org/10.1016/j.envpol.2018.11.055			x	x	x				x		Yes
Grintzalis et al., 2019	https://doi.org/10.1080/17435390.2019.1577510			x	x					x		No
Zhang et al 2020	https://doi.org/10.1016/j.chemosphere.2019.124563			x			x				x	No
Wu et al 2019b	https://doi.org/10.1016/j.aquatox.2019.105350		x				x				x	No
Liu et al 2020 a	https://doi.org/10.1016/j.envpol.2019.113506			x			x			x	x	No
Liu et al., 2020 b	https://doi.org/10.1016/j.chemosphere.2020.126065			x			x				x	No
Frankel et al., 2020	https://doi.org/10.1039/c9en01236b		x		x					x		Yes
Zhang et al., 2019	https://doi.org/10.1007/s11356-019-05031-2			x			x			x	x	No
Fadare et al., 2020	https://doi.org/10.1021/acs.est.0c00615		x		x					x	x	No
Zhang et al., 2020	https://doi.org/10.1016/j.envpol.2019.113451			x	x	x				x		No
De Felice et al., 2019	https://doi.org/10.1016/j.chemosphere.2019.05.115			x			x			x		No
Saavedra et al., 2019	https://doi.org/10.1016/j.envpol.2019.05.135			x	x	x				x		No

De Felice et al., 2022	https://doi.org/10.1016/j.envint.2022.107264	x		x	x	x	x	No
Ma et al., 2021	https://doi.org/10.1016/j.bbrc.2021.10.062	x		x			x	No
Nogueira et al., 2022	https://doi.org/10.1016/j.scitotenv.2021.151360	x		x		x	x	No
Pochelon et al., 2021	https://doi.org/10.3390/environments8100101		x			x		No
Verdu et al ., 2022	http://dx.doi.org/10.1016/j.scitotenv.2022.153063	x		x		x		Yes

Table S2. Minimum reporting information (MRI) list

To be filled in where applicable and included in the Supplementary information of the paper

**Studied nanoplastics**	**Test organism**	**Sample preparation**	**Exposure conditions**	**Data quality**
**Very important criteria**				
Polymer chemical composition	test species	removal of particle impurities and additives	Exposure duration	Number of replicates
Particle origin	strain identification	dispersion medium	exposure medium	statistical method/model
primary particle size	sex	medium properties (pH, O2, OM, conductivity)	Static OR flow through	Negative control
particle morphology	length	particle dispersion aids	Tested concentrations	Reference (natural) particle
particle primary surface charge	body weight		-	Test quality criteria
particle primary surface functionalization	age		Measured toxicity endpoints	
particle (suspension) chemical impurities	growth stage		Toxicity values/effect	
particle (suspension) additives				
**Medium importance criteria**				
particle specific surface area		additional test medium properties	Toxicity recording frequences	Reference chemical
		leached chemicals		Nanoplastics background
		-		
